# Philippine crimes of dissent: Free speech in the time of COVID-19

**DOI:** 10.1177/1741659020946181

**Published:** 2020-07-30

**Authors:** Jeremiah Joven B Joaquin, Hazel T Biana

**Affiliations:** Department of Philosophy, De La Salle University, Philippines

## The *Bayanihan* to Heal as One Act

The novel coronavirus disease, COVID-19, has become a global health crisis, where, as of June 13, 2020, hundreds of thousands have died and millions of people have been infected. In the Philippines, this translates into more than 25,000 infected, and over a thousand deaths. In a country with a dual and decentralised healthcare system, wherein only a part of the health premiums are subsidised by the government, the pandemic is a huge health challenge because of resource and capacity limitations.

As a response, the Philippine government has undertaken measures to curb the spread of the virus. This includes travel restrictions, various levels of community quarantines and the *T3* (test, trace and treat) program. To fully implement these measures, President Rodrigo Duterte signed into law Republic Act No. 11469 or the *Bayanihan to Heal as One* Act on March 25, 2020. This act declares the pandemic as a national emergency and authorises the president to exercise emergency powers necessary to ensure public health and safety (Congress of the Philippines, 2020a). Though the *Bayanihan* Act is commendable for its provisions on the remuneration for healthcare front-liners, a prohibition on the hoarding of basic necessities, and cash and non-cash relief for indigent and unemployed citizens, it still includes a highly questionable provision on the criminalisation of fake news.

## The false information provision

Section 6f of the *Bayanihan* Act criminalises people who make and spread false information (on social media and other platforms). Accordingly, people who proliferate information that have ‘no valid or beneficial effect on the population, and are clearly geared to promote panic, chaos, anarchy, fear and confusion’ shall be imprisoned for up to 2 months or be fined for up to one million pesos (approx. US$ 25,000.00) (Congress of the Philippines, 2020a).

In less than a month since its implementation, 47 persons were nabbed for the alleged violations of the provision (Caliwan, 2020). Among them is the celebrated Cebu-based artist and scriptwriter, Maria Victoria Beltran. Due to her COVID-19 satirical post on social media, where she wrote: ‘9,000+ new cases (All from Zapatera) of COVID-19 in Cebu City in 1 day. We are now the epicentre in the whole Solar System’, Beltran was threatened by the city mayor, put in jail and even had to post bail amounting to PhP 42,000.00 (approx. US$ 800.00). She was ‘arrested by the Philippine National Police in the dark hours of midnight, interrogated, tied to a chair like a dog. . . and held *incommunicado* for 16 hours’ (Panti, 2020).^1^

## A violation of the Philippine constitution

A bigger issue with this specific provision is that it seems to be at odds with the basic civic freedoms guaranteed by the governing 1987 Philippine Constitution. According to the Bill of Rights of the Constitution, ‘No law shall be passed abridging the freedom of speech, of expression, or of the press, or the right of the people peaceably to assemble and petition the government for a redress of grievances’ (Constitutional Commission of the Philippines, 1987). The fake news provision of the *Bayanihan* Act seems to violate this.

The Philippine Commission on Human Rights has weighed in on the issue and has stated that ‘A fully functional democratic society should be able to allow the reasonable exercise of free speech and expression as a means to participate in matters concerning public life. Arrests should never be made as a default response to dissent’. Moreover, ‘Human rights cannot be suspended even during public emergencies. Restrictions to freedoms are also bound by the parameters set by human rights law and should never lead to their abrogation’ (Panti, 2020).

Perhaps, the *Bayanihan* Act’s provision on fake news is grounded on some sound principle. With the Philippine public exposed to various information, being privy to facts is vital for surviving the pandemic. As such, curtailing free speech might be needed to avoid unnecessary panic. Whatever the intention of the makers and spreaders of fake news may be, so long as the information causes the public undue ‘panic, chaos, anarchy, fear and confusion’ during the pandemic, then it is just plain wrong and needs to be penalised. This provision of the *Bayanihan* Act seems to present tension between preserving free speech, on the one hand and ensuring public safety, on the other hand. We think, however, that this tension is false and specious.

## The not-so-urgent *Bayanihan* amendment and the ever-so-urgent anti-terror bill

The fake news provision of the *Bayanihan* Act ostensibly aims to protect the general public but actually facilitates the government’s crackdown on dissent; that is, this piece of emergency legislation is being used to further wider political suppression. This seems to be a trend all over Southeast Asia as COVID-19 ‘has provided a handy excuse for a clampdown on free speech’ (Sochua, 2020). To this end, UN High Commissioner for Human Rights Michelle Bachelet cautioned that it seems that ‘some governments were taking advantage of emergency powers. . . to stifle dissent’ (Perelman, 2020). Like the aforementioned Beltran case, such crackdowns in the Philippines seem to be directed to ‘political foes, human rights defenders and journalists’ (Perelman, 2020).

Before the original *Bayanihan* Act expired on June 25, 2020, the Philippine Senate drafted the *Bayanihan to Recover as One* Bill (or *Bayanihan* 2), which is supposed to be just a relief bill that focuses on re-starting the economy through budget provisions and repeals the fake news provision of the original *Bayanihan* Act. On June 4, 2020, Senator Franklin Drilon reminded law enforcers, arrests could no longer be made on the basis of fake news as the original law had lapsed (Bordey, 2020). President Duterte, however, did not certify *Bayanihan* 2 as urgent, and since the Senate had already adjourned its session, this leaves the country with no specific law on programs and interventions to cover itself during the pandemic

While *Bayanihan* 2 was not passed into law, the new Anti-Terrorism Act of 2020 was on June 3, 2020. Certified as urgent by the government, the Anti-Terrorism Act is an amendment to the Human Security Act of 2007 and was transmitted to the President for his signature several days before the *Bayanihan* Act lapsed its effectivity (Congress of the Philippines, 2020b). The new law creates an Anti-Terrorism Council, whose members are primarily composed of the executive branch of the government. The council decides who the terrorists are and permits their warrantless arrest and detention for up to 30 days. The council may also allow the wiretapping and surveillance of suspected individuals for extended periods of time (Congress of the Philippines, 2020b). Like the fake news provision of the *Bayanihan* Act, the Anti-Terrorism law may curtail freedom of speech and expression; the latter law pushes the envelope further as it may also encroach on one’s freedom of association and the right to due process.

Alarmed by the human rights abuses that the Anti-Terrorism law may bring, the International Coalition for Human Rights in the Philippines (ICHRP) Chairman Peter Murphy stated that the government seems to be ‘giving priority to repressive legislation but remains bungling in addressing the pandemic. We have seen too many deaths both from the virus and the violence President Duterte has unleashed against his people’ (ul Khaliq, 2020).

All over the country, mass protests have been held to question the timeliness and purpose of the Anti-Terror Law. More than a thousand people wore face masks, observed social distancing, while gathering in protest on June 12, 2020, the 122nd anniversary of Philippine independence from Spanish rule. In Iligan City though, 16 youth protesters were arrested and detained for allegedly violating physical distancing measures and quarantine protocols during their assembly that lasted for less than 10 minutes (Talambong, 2020).

## Conclusion

The Philippine government’s intention to criminalise fake news might be motivated by the noble goal of ensuring public safety in the time of the COVID-19 pandemic. The non-prioritisation of *Bayanihan* 2 and the passage into law of the Anti-Terrorism Bill, however, may put this motivation into question. At a time when infection rates have steadily been increasing in the Philippines despite having one of the longest and strictest lockdowns, one wonders why the government seems to prioritise curbing terrorism over the pandemic. Perhaps, this is for a genuine security concern. Perhaps, this is to silence its ardent critics. Perhaps, this is a red herring to mask its incompetence in handling the health crisis. Whatever the reason may be, it is a no-win situation for Filipinos. While their lives are still at risk due to the threat of COVID-19, their most cherished democratic right of free speech also hangs in the balance.

## Notes

1. For her commitment to free speech, Beltran was recognised by international broadcasting network Deutsche Welle (DW) and was one of the 17 recipients of 2020 DW Freedom of Speech Award last May 6, 2020 (Mayol, 2020).
